# Associations Between Addictive Behaviors, Individual Characteristics, and the Use of Gambling Services Within the World of Gaming: Cross-sectional Survey Study

**DOI:** 10.2196/29077

**Published:** 2022-04-22

**Authors:** Mark Kisch, Anders Håkansson

**Affiliations:** 1 Division of Psychiatry Department of Clinical Sciences Lund University Lund Sweden; 2 Malmö Addiction Center Region Skåne Malmö Sweden; 3 Clinical Research Unit/Clinical Gambling Disorder Unit Malmö Addiction Center Region Skåne Malmö Sweden

**Keywords:** gambling disorder, gaming disorder, behavioral addiction, mental health, gambling, gaming, addiction, behavior, cross-sectional, online survey, age, gender

## Abstract

**Background:**

Gambling within the world of gaming is an emerging phenomenon that may share common conceptual characteristics with traditional forms of gambling. The current literature suggests a higher degree of problematic behaviors in this gambling pattern, but studies are few, prompting for further research regarding individual characteristics and comorbid conditions associated with this activity.

**Objective:**

The aim of the study is to investigate correlations between the use of gambling services within the world of gaming and individual characteristics and addictive behaviors including problem gambling.

**Methods:**

A cross-sectional web survey was distributed to an existing panel of online respondents in Sweden. A total of 2001 respondents were included. Chi-square and Mann-Whitney *U* tests, followed by a logistic regression, were used in order to determine independent variables associated with gambling in the context of gaming.

**Results:**

A total of 2.9% (58/1984) of respondents reported past-year gambling within gaming. Significant associations were found with male sex, younger age, history of treatment-seeking for alcohol problems, and higher Gaming Addiction Scale scores.

**Conclusions:**

The demonstrated findings strengthen previously found associations between gambling in gaming and younger age, male sex, and problematic gaming behaviors. Additionally, the association with a history of treatment needs for alcohol problems adds to the previous impression of increased problem severity and comorbidity in within-gaming gamblers.

## Introduction

Gambling disorder is a psychiatric diagnosis describing a problematic gambling pattern leading to substantial impairment or mental distress [1]. The prevalence of either problem gambling or gambling disorder has been estimated to be between 0.1% and 5.8%, [2], and risk factors for problem gambling include male sex, young age, substance use, poor mental health, low level of formal education, and low socioeconomic status [3,4]. Gambling disorder can be treated in psychological therapies in the form of cognitive behavioral therapy [5]. Since the 1980s, the world has seen an incomparable growth in global commercial gambling, which is suggested to be due to changes in attitudes toward legal gambling or the increasing presence of internet and mobile devices and other nontraditional platforms that provide gambling services [6].

A number of harms have been described to be associated with a history of gambling disorder [7]. Financial harm is a dominant theme that may include debts and personal bankruptcy, affecting the gambler, relatives, and society as a whole. Furthermore, there are documented high rates of comorbid psychiatric disorders among gamblers. A meta-analysis performed on 36 studies documented comorbid, current psychiatric disorders in 74.8% (95% CI 36.5-93.9) of treatment-seeking gamblers [8]. The most prominent types are nicotine dependence, substance use disorders, mood disorders, and anxiety disorders [9]; suicidal ideation, suicide attempts [10], and completed suicides [11] are also seen.

An increasing proportion of gamblers engage in online platform activities [12], characterized by high availability, a short time between betting and outcome, light and sound effects, uninterrupted gaming sessions, variable stake sizes, near miss features, anonymity, and illusion of control [13]. As found in an annual report from the Gambling Commission in Great Britain, 18% of the respondents had gambled online in the past 4 weeks [14]. In addition, problem gambling is more common in internet gambling compared to noninternet gambling [15]. When compared to noninteractive gamblers, interactive gamblers are more likely to be younger, male, educated, and part of a group household. Interactive gamblers also tend to gamble on more activities and bet higher amounts [16]. In Sweden, a large majority of treatment-seeking gamblers are involved in online gambling activities [17]. Younger adults may represent a vulnerable subgroup with an increased risk to experience gambling problems, and this appears to be independent of gambling modality or degree of gambling participation [18,19]. The comparatively increased risk-taking and poor consequence thinking in this group has been suggested as a possible explanation [20].

The phenomenon of digital- and video-gaming is increasingly highlighted with respect to its addictive properties and problematic behaviors with negative outcomes [11,21]. Gaming disorder has appeared in the World Health Organization’s *International Classification of Diseases* since 2018. The diagnosis is characterized by an at least 12-month period of impaired control of gaming, reprioritization of daily activities and interests that allows more time to spend on gaming, and continued gaming despite negative consequences, along with a clinically significant loss in function in important life areas [22]. In the *Diagnostic and Statistical Manual of Mental Disorders, Fifth Edition* (DSM-5), internet gaming disorder is recognized as a condition warranting more clinical research before it might be listed as a formal disorder [1].

Computer gaming is a common phenomenon, especially in the lower age categories where the majority is engaged in this activity in some way [23]. Concerning the prevalence rate of internet gaming disorder among adolescents, data indicate that this is on the rise and has been estimated to be 1.6% in European adolescents and significantly more common in boys [24,25]. Due to the limited amount of longitudinal studies, causes and consequences in the field of pathological gaming are not fully investigated [26,27]. However, it seems that, for example, lower psychosocial well-being, including lower social competence and self-esteem, is an antecedent rather than a consequence of pathological gaming. Loneliness, on the other hand, turns out to be both a cause and consequence in this manner [28]. Other risk factors for developing internet gaming disorder include greater amounts of gaming participation and impulsivity traits, while depression, anxiety, and lower school grades should be seen as consequences [29]. In addition, general population data show that daily playing of computer games may be associated with a marked increase in the risk of problem gambling, regardless of gender [23].

In the context of gaming, specific features appear to overlap structurally and psychologically with conventional types of gambling for money [30]. Built-in gambling elements in games can take the form of loot boxes purchasable for real money, randomly generating virtual items that can be either cosmetic assets (ie, skins) or performatively advantageous in the game in question [31,32]. Virtual currencies can be sold or cashed out for real money, which creates a situation similar to traditional forms of gambling [30]. These convergences between gambling and gaming are often facilitated by digital microtransactions [33-35]. Notably, the authors label monetization technologies in games such as loot boxes as predatory due to their properties to encourage escalating player spending [36].

According to a recent Danish national survey, more than half of the gaming adolescents in the sample, with a male predominance, had engaged in loot box activity at some time [37]. Additionally, a positive correlation was identified between loot box engagement and problem gambling. In a Swedish population study, at-risk gambling was considerably more common in those who had bet on loot boxes [23]. Similar correlations have been recognized in the context of skin betting (gambling with items within the game that have value to the player’s character) among adolescents [38]. Loot box involvement, giving rise to more problematic gaming behaviors, also has been indirectly linked to mental distress [39], and links between the amount spent on loot boxes and the degree of gambling problems have been acknowledged [32]. Loot box users are more likely to be young, employed, low-educated, and have an average household income [40].

Given these concerns, an important challenge worth emphasizing is the lack of international consensus for where the boundaries should be set for gambling-in-gaming activities to meet legal definitions on gambling [41-43]. These blurred boundaries between gambling and gaming call for more research addressing individual characteristics associated with the use of gambling services in the context of gaming.

Overall, the phenomenon of gambling within computer games has been increasingly highlighted, but studies of its correlates in the population are still few. Additionally, uncertainty remains about how this habit is associated with other health hazards, such as poor mental health and more traditional addictive disorders to tobacco, alcohol, and drugs, over and above the need to study it in the context of problem gambling and problem gaming. Therefore, the aim of this study is to, on the basis of responses from an electronic self-response questionnaire, investigate correlations between the use of gambling within gaming services while controlling for a number of sociodemographic, substance use, and mental health variables, as well as established measures of problem gaming and problem gambling.

## Methods

### Study Sample

A targeted Swedish segment of Userneeds web panel, consisting of a total of 115,000 individuals aged 16 years and older, was derived with the aim of accurately reflecting Sweden’s general population in terms of sex and age distribution. From the way that data collection took place, it follows that it is not possible to determine how many individuals in fact were contacted in order to reach the intended number of respondents.

Out of a total of 2124 respondents, 116 individuals did not meet the primary inclusionary criteria which were formulated as having completed all the questions of the survey. Additionally, 7 sets of answers could be paired with possible doublets, originating from the same IP addresses and indicating duplicate answers. Consequently, the second round of these responses were excluded from the material, resulting in a final sample of 2001 completed surveys. Of these, only those responses that were consistent with the defined outcome measures for each question were used for further analysis in this study. This means that respondents who reported answers “don’t know and/or don’t want to answer” on questions where these answer options were available were designated as missing cases and hence not included in numerical calculations. The number of missing cases was different for each question but amounted at most to 48, so the total number of respondents with full data included in the final statistical analysis was n=1953.

### Procedure

Recruitment of participants was carried out using the electronic questionnaire services of the company Userneeds, resulting in a cross-sectional study design. More specifically, a quantitative web survey was distributed to the Swedish segment of the company’s multinational web panel consisting of preexisting survey respondents who had voluntarily signed up as members and provided Userneeds with personal information in order to regularly receive email invitations to participate in online surveys. The invitations sent out to this study contained written information regarding the anonymity of participation as well as on the topic of the survey referred to as “gambling, addiction to gambling, and mental behavioral and substance use disorders.” In order to start responding to the questionnaire, participants were required to give their written electronic consent. Data collection took place over 17 days in September 2019 and was scheduled to cease when 2000 individual responses were achieved. As a general reward for completing surveys distributed from Userneeds, responders receive credits equivalent to approximately one euro in the company’s bonus system.

As mentioned above, the survey contained a number of questions focusing on gambling behaviors but beyond that also intended to capture the sociodemographic background of respondents as well as comorbidities in the form of mental illness and other addictions. Based on the replies to whether or not the respondent in the past year had engaged in gambling services within computer games, the material was divided into 2 main groups: those who had engaged in this activity (n=58) and those who had not (n=1926). A total of 17 respondents answered “don’t know and/or don’t want to answer” on this question and were consequently not included in the study. The 2 groups with valid answers, however, were studied separately and compared in respect of correlates by means of variables obtained from the survey responses.

### Ethics Approval

From the same overall research project, one study has been published and another is under review addressing other aspects of gambling and behavioral addictions [44]. The Ethics Review Authority, Sweden, reviewed the ethics application (number 2019-04176) and found the study did not formally require ethical permission as no personal data were used and also expressed that it had no ethical concerns with the study. Respondents enrolled in the study participated on a completely voluntary basis and gave their written consent in order to be able to start answering the questionnaire.

### Measures

#### Sociodemographic Variables

Gender was categorized into the binary groups of male and female; those who preferred not to answer the question of gender identity were excluded from the study. Age was stratified into 6 brackets of age ranges (16-19 years, 20-24 years, 25-29 years, 30-39 years, 40-49 years, and 50 years and older). Monthly income was stratified into 10 brackets measured in Swedish krona (SEK) from <10,000 SEK (US $1000) to >50,000 SEK (US $5000). Level of education was categorized on the basis of ever having attended university or not, regardless of obtained degree. Finally, the living status was spaced into 5 categories: single with children living at home, single without children living at home, living with a husband/wife or partner with children at home, living with a husband/wife or partner without children, or living with parents.

#### Comorbidity Variables

In order to identify serious mental illness among the respondents, the Kessler Psychological Distress Scale (K6) was integrated into the survey. K6 is a 6-item self-reported tool designed to screen for mood and anxiety disorders over the past 6-month period. Each question in the inventory investigates to what extent distinct feelings of nervousness, hopelessness, restlessness, depressiveness, lethargy, and worthlessness have been experienced. The responses are consequently coded with scores depending on the stated frequency of each question where 0=none of the time, 1=little of the time, 2=some of the time, 3=most of the time, and 4=all of the time. This system results in a total score range extending from 0 to 24 [45]. A categorical variable was defined by a summated cutoff score of ≥13, having a sensitivity of 0.36, and a specificity of 0.96 for detecting serious mental illness [45]. In the collected material for this study, 24 individuals had missing answers on one or two K6 items. In these cases, the individuals’ median values of the available items were used to calculate their complete K6 scores.

In addition to K6, the survey included questions about having a history of seeking treatment for either psychological distress or problems with alcohol, illicit drugs, or prescription drugs. A question about daily use of nicotine, as in smoking or snuff, was also included.

#### Problem Gambling Severity Index

The 9-item scale [46,47] of the Problem Gambling Severity Index (PGSI) consists of questions asking about how frequently the responder engage in behaviors related to problem gambling, thus aiming to determine problem gambling severity. Each answer is scored from 0 to 4 based on a Likert scale consisting of the following response options: 0 (never), 1 (sometimes), 2 (most of the time), and 3 (almost always). Hence, depending on the total score, ranging on a continuum from 0 to 27, responders can traditionally be classified as being a nongambler or nonproblem gambler (0 points), low-risk gambler (1-2 points), moderate-risk gambler (3-7 points), or problem gambler (≥8 points). In this study, however, PGSI scores were used in a continuous manner rather than with the intention of categorizing participants into certain gambling profiles.

In order to assess gambling behaviors more generally, the survey contained questions on whether respondents have engaged in other forms of past-year gambling activities (online casino, physical casino, online horse betting, physical horse betting, online sports betting, physical sports betting, online poker, physical poker, online bingo, and physical gambling machines).

#### Gaming Addiction Scale

The Gaming Addiction Scale (GAS) is a 7-item questionnaire documented to have high reliability and generate consistent results across various samples [48]. The questions included in the GAS are based on DSM-based criteria for internet gaming addiction, and response options were subsumed according to a Likert scale ranging from 1=never to 5=very often. Assessing the past 6-month period, the questions asked intend to measure gaming-related preoccupation, tolerance, escapism, difficulties of quitting, withdrawal signs, social conflicts, and problems [48]. In the same way as for the PGSI, GAS scores were used as continuous measures in this study.

### Statistical Analysis

The statistical analyses were conducted using SPSS (version 26, IBM Corp). First, the group of responders that in the past year had engaged in gambling services within computer games was compared with the group who had not engaged in this activity with regard to 20 categorical variables. These comparisons were made using chi-squared tests and included gender; age; monthly income; living status; level of education; serious mental illness according to K6; history of treatment-seeking due to psychological distress, alcohol problems, or problems with narcotics/drugs; and daily use of nicotine as well as past-year engagement in other traditional gambling activities. The total scores of the variables PGSI and GAS were kept as continuous scales rather than classifying tools and compared between the groups in terms of median values using nonparametric Mann-Whitney *U* tests. In order to investigate adjusted correlates of the use of gambling services within computer games, variables that according to the above-mentioned statistical models demonstrated statistically significant differences between the groups (*P*<.05) could enter a binary logistic regression. In order to stay within the recommended range of minimum events per predictor variable [49], the variables measuring other forms of gambling activity in the past year were not included in the logistic regression analysis. That way, a total of 10 variables entered the final analysis, which meant an events per predictor variable of 5.8. Results of the logistic regression were reported as odds ratios with 95% confidence intervals.

## Results

Among the 2001 responders who completed the survey, 17 individuals answered “don’t know and/or don’t want to answer” on whether they had engaged in gambling services within computer games in the past year. Among the remaining 1984 responders with valid answers on this question, 2.9% (58/1984) reported engagement in this activity during the given period.

The unadjusted comparison between gamblers in computer games and the remaining respondents of the sample showed that those who participated in gambling services within computer games in the past year were significantly (*P*<.05) more likely to be male, younger, and single or living with their parents, as well as to have a lower degree of education. In terms of comorbidity variables, significant associations were found between gambling in gaming and serious mental illness according to K6; treatment-seeking for problems with alcohol, illicit drugs, and prescription drugs; and daily use of nicotine. Involvement in gambling services within the world of gaming was significantly associated with higher past-year engagement in other forms of gambling activities (online casino, physical casino, online horse betting, physical horse betting, online sports betting, physical sports betting, online poker, physical poker, online bingo, and physical gambling machines) as well as higher median scores on the PGSI (8 vs 0, *P*<.001) and GAS (21.5 vs 7, *P*<.001; Table 1).

**Table 1:** Comparison between gamblers in computer games and the rest of the sample using chi-square and Mann-Whitney analyses (n=1986).

			Past-year gambling within computer games (n=58), n (%)		No past-year gambling within computer games (n=1926), n (%)		*P* value		Missing (“don’t know or don’t want to answer”)
Male gender			45 (77.6)		930 (48.3)		<.001		2
**Age (years)**			—^a^		—		<.001		0
	16-19	9 (15.5)		49 (2.5)		—		—	
	20-24	13 (22.4)		98 (5.1)		—		—	
	25-29	12 (20.7)		178 (9.2)		—		—	
	30-39	8 (13.8)		352 (18.3)		—		—	
	40-49	13 (22.4)		477 (24.8)		—		—	
	>50	3 (5.2)		772 (40.1)		—		—	
**Monthly income (SEK)**			—		—		.19^b^		0
	<10,000	12 (20.7)		175 (9.1)		—		—	
	10,000-15,000	6 (10.3)		182 (9.4)		—		—	
	15,000-20,000	5 (8.6)		179 (9.3)		—		—	
	20,000-25,000	4 (6.9)		199 (10.3)		—		—	
	25,000-30,000	8 (13.8)		308 (16.0)		—		—	
	30,000-35,000	8 (13.8)		302 (15.7)		—		—	
	35,000-40,000	4 (6.9)		225 (11.7)		—		—	
	40,000-45,000	2 (3.4)		127 (6.6)		—		—	
	45,000-50,000	1 (1.7)		66 (3.4)		—		—	
	>50,000	8 (13.8)		163 (8.5)		—		—	
**Living status**			—		—		<.001		0
	Single with children at home	5 (8.6)		103 (5.3)		—		—	
	Single without children at home	17 (29.3)		496 (25.8)		—		—	
	With husband/wife or partner and children	14 (24.1)		574 (29.8)		—		—	
	With husband/wife or partner and no children	11 (19.0)		659 (34.2)		—		—	
	Living with parents	11 (19.0)		94 (4.9)		—		—	
Attended university			19 (32.8)		1084 (56.3)		<.001		0
Serious mental illness on the K6^c^ (≥13 points)			18 (31.0)		170 (8.9)		<.001		10
Need of treatment for psychological distress			27 (47.4)		695 (36.6)		.10		27
Need of treatment for alcohol problems			13 (23.6)		66 (3.4)		<.001		14
Need of treatment for illicit or prescription drug problems			8 (14.3)		30 (1.6)		<.001		8
Daily use of nicotine (smoking or snuffing)			18 (32.1)		301 (15.7)		.001		8
**Past-year gambling activities**			—		—		—		—
	Casino, online	26 (47.3)		133 (6.9)		<.001		9	
	Casino, physical	16 (28.1)		57 (3.0)		<.001		6	
	Horse betting, online	25 (43.1)		248 (12.9)		<.001		8	
	Horse betting, physical	16 (29.1)		196 (10.2)		<.001		13	
	Sports betting, online	26 (47.3)		303 (15.8)		<.001		8	
	Sports betting, physical	21 (36.8)		217 (11.3)		<.001		6	
	Poker, online	19 (33.3)		49 (2.5)		<.001		4	
	Poker, physical	12 (21.8)		54 (2.8)		<.001		5	
	Bingo, online	25 (44.6)		84 (4.4)		<.001		6	
	Gambling machines, physical	22 (37.9)		81 (4.2)		<.001		1	

^a^Not applicable.

^b^Linear by linear association.

^c^K6: Kessler Psychological Distress Scale.

In logistic regression of the statistically significant variables (except the variables on past-year engagement in other forms of gambling activities), gambling in gaming remained significantly associated with male sex, younger age groups, treatment-seeking for alcohol problems, and higher median scores on the GAS (Table 2).

**Table 2:** Potential correlates of gambling within the world of gaming (logistic regression including variables with significance in bivariate analyses [n=1953]).

	Odds ratio (95% CI)
Male gender	3.30 (1.53-7.12)
Age	0.76 (0.60-0.97)
Living status	0.87 (0.65-1.15)
Attended university	0.65 (0.33-1.31)
Serious mental illness on the K6^a^ (≥13 points)	0.96 (0.42-2.18)
Need of treatment for alcohol problems	3.08 (1.09-8.69)
Need of treatment for narcotics or addictive drug problems	0.59 (0.16-2-20)
Daily use of nicotine (smoking or snuffing)	1.39 (0.63-3.03)
PGSI^b^ median score	0.98 (0.91-1.05)
GAS^c^ median score	1.27 (1.19-1.37)

^a^K6: Kessler Psychological Distress Scale.

^b^PGSI: Problem Gambling Severity Index.

^c^GAS: Gaming Addiction Scale.

## Discussion

### Principal Findings

The aim of this study was to determine potential correlates of the use of gambling services within the world of gaming, focusing on sociodemographic variables, co-occurring mental health problems and substance use, and gaming addiction and problem gambling levels. In the study sample, which had a cross-sectional design and was intended to reflect the general population of Sweden, 2.9% (58/1986) of respondents (aged 16 years and older) reported they had engaged in gambling services within computer games in the past year. After controlling the variables for one another, however, significant associations were found between gambling in gaming and male gender, younger age, history of treatment-seeking due to alcohol problems, and higher scores on the GAS. In the unadjusted analyses, individuals involved in gambling within gaming had a markedly higher problem gambling score. However, no independently significant association was found between gambling in gaming and problem gambling when controlling for other relevant variables.

In terms of the prevalence of gambling in gaming, previous research conducted in the area has quite unanimously targeted subgroups of the population such as adolescents or excessive gamers, making our findings one of the first measures of prevalence in the general population. The only comparable figure found was 1.3%, calculated from a recent German sample of internet users [40].

This study examined a series of sociodemographic variables as potential correlates of the involvement in gambling in gaming. Of these, gambling in gaming could be significantly associated with male sex and younger age in the final logistic regression model. More specifically, among the respondents who reported they had engaged in gambling-like activities within computer games the past year, 78% were male and 59% were aged 29 years or younger. Corresponding figures for the compared group were 48% and 17%, respectively. These results were consistent with findings from the limited studies conducted in the area [39,40]. Considering the young male predominance in the context of excessive gambling and gaming in general [23,49], it was not surprising that the distribution of age and sex in the field of convergence between these activities seems to follow the same pattern.

Since poor mental health and substance use problems are commonly co-occurring with problem gambling [3,4], this study intended to investigate whether these associations exist in the case of individuals who engage in gambling in gaming. Li and colleagues [39] had previously indirectly, through the adverse effects of excessive gaming, linked loot box involvement to higher levels of mental distress, yet no significant association was found in our study between gambling in gaming and serious mental illness in the adjusted analysis. Here, one should bear in mind the low sensitivity (0.36) of the K6 scale’s ability to detect serious mental illness [45], but on the other hand, a history of treatment-seeking for psychological distress showed no significant association with gambling in gaming in the unadjusted or adjusted analysis, indicating that such a direct association may not exist. Instead, a history of treatment-seeking for alcohol problems was 8 times as common in the group that had engaged in gambling in gaming. To the best of the our knowledge, previous data for comparison is lacking in this regard. Moreover, drinking alcohol while gambling increases the risk of developing gambling problems and vice versa [50], and comorbidity between problem gambling and alcohol use disorders is high [51]. Thus, as traditional problem gamblers share symptom characteristics and to some extent neurobiological and psychological pathophysiology with substance use disorders [52,53], it is worth investigating if this is also the case for the gambling in gaming phenomenon.

Comparing median scores on the GAS between the group that reported they had engaged in gambling in gaming the past year versus the group that had not, significantly higher scores were found in the former group, both in the unadjusted and adjusted analyses. More specifically, respondents who had engaged in gambling in gaming displayed a median score of 21.5 while the group for comparison scored 7, which according to the adopted scale model is the lowest possible total points, indicating absence of gaming-related problems in a very large portion of the general population. This should be read as such criteria largely based on what have been suggested to define internet gaming disorder according to the DSM-5 being found to be fulfilled to a greater extent in the group that had engaged in gambling in gaming. Given that the objective of the study, in this aspect, was to look for associations between gambling in gaming and gaming-related problems in general, continuous scoring was an appropriate approach that would presumptively detect such a link if it existed. At the same time, it follows that the use of the GAS scale as a continuous variable did not claim to detect and address gaming disorder as a psychiatric diagnosis among the respondents, given that this would require demarcations on how many criteria of the 7 items, and to what extent, each respondent met [48]. The positive association found between gambling in gaming and gaming-related problems, however, is concurrent with the discoveries of the few studies conducted in the area, although different screening tools had been used and the monetization type of the games had in these cases been limited to loot boxes [39,40].

In terms of the association between gambling in gaming and problem gambling, previous research has been fairly unanimous that such a link seems to exist [37,38]. Here, however, while the uncontrolled analyses showed a statistically significant and large difference in the PGSI median scores (8.0 vs 0.0) between the respondents with and without engagement in gambling in gaming, these figures did not retain their significance in the final linear regression analysis model. A likely explanation may be that problematic gambling behavior was more strongly explained by elevated GAS scores than PGSI scores, with reference to the previously cited, indirect relationship between daily gaming and problem gambling [23]. In the context of multidimensional gambling behaviors, however, all other forms of gambling participation in the past year tested for in the unadjusted analysis were more common among respondents who had also engaged in gambling in gaming. Wardle [38] concluded in a recent study that while skin betting as an isolated phenomenon was not significantly associated with at-risk gambling, in combination with other gambling activities it was highlighted as a considerable risk factor for developing problematic gambling behaviors by underpinning the same behaviors. Thus, it is theoretically possible that such multifactorial explanation models exist, which prompts for more research in the area.

### Implications

This study strengthens the limited scientific basis for the associations previously found between participation in gambling-like activities within computer games and male gender, younger age, and problematic gaming behaviors. However, the demonstrated association between gambling in gaming and a history of treatment-seeking for alcohol problems can be considered a new supplement in the emerging description of the profile characteristics of individuals who engage in this overlapping activity between gambling and gaming. Additionally, this newfound link may provide hints about underlying dependence mechanisms or risk factors in this field of fusion between gambling and gaming, prompting for closer investigation of the associations with substance use disorders as well as possible bidirectional relationships.

As no significant associations could be found between gambling in gaming and serious mental illness or problem gambling, which were findings that to varying degrees differed from existing data, these relationships must be explored further. Since excessive gaming and in-game gambling services are predominantly a problem in the younger population, longitudinal study designs are needed in order to be able to monitor whether involvement in and normalization of such activities at a young age could predispose to gambling problems once these individuals reach legal age. Likewise, given that gambling in gaming is most prevalent in younger segments, this is an urgent aspect that needs to be further investigated for the basis of prevention work.

### Strengths and Limitations

The main strength of this study, given its exploratory intentions, is that it is based on a broad study sample with participants recruited from the general population rather than from psychiatric clinical settings where this kind of screening for correlates could be deceptive.

The limitations of this study largely depend on data acquisition–related aspects, given that the collected material consisted of self-estimates and self-responses, and it is a likely assumption that web panel members with a greater interest in or experience of with gambling, mental behaviors, and substance use may have been more prone to participate in the study after the topic was presented in the invitation mailings. Whether online behaviors of individuals who are part of a web panel and recurrently enroll in online market surveys differ from the general population cannot be ruled out. These settings may have contributed to a selection bias, skewing the studied population toward a higher degree of problematic behaviors in several respects that the study intended to investigate. In the light of the findings, however, these circumstances are most important to bear in mind when drawing conclusions about associations between gambling and alcohol issues. On the other hand, the self-administered online questionnaires have the advantage of decreasing the impact of social desirability bias, as respondents were anonymized toward the authors and final scores and potential study implications were withheld.

Another possible limitation of the study that may have impacted the results is that the study did not include individuals younger than age 16 years, a group where, considering the current state of knowledge, involvement in gambling services within computer games can be assumed to be high. This means that the estimated 3% prevalence of past-year engagement in gambling in gaming could be on the low side and should consequently not be taken for granted or applied to other populations, especially because online habits may differ considerably between different countries. However, it is also possible that the age bracket comprising those aged 16-19 years may have encompassed a minor number of participants aged 16 or 17 years, whose hypothetical impact on the results of the study is somewhat difficult to eradicate since the items of the PGSI instrument in many ways undertake legal age and responsibility for one’s own finances.

Despite the relatively high rates of engagement in gambling in gaming, the absolute number of respondents belonging to this subgroup was low (n=58) and all of the intended variables could not be included in the overall logistic regression analysis model. Although this can be considered a limiting factor of this study, our findings may serve as guidance for future studies when sample sizes are to be determined in order to allow for more elaborate and multifactorial statistical procedures.

Finally, although it was not the aim of the study, it is noteworthy that this study design does not allow for the determination of temporality aspects between gambling in gaming and the associations found. In order to establish such time relationships and ultimately understand underlying pathological pathways, further research of longitudinal and prospective approaches is required.

### Conclusions

The demonstrated findings of this study have strengthened previously found associations between gambling in gaming and younger age, male sex, and problematic gaming behaviors. Additionally, an association was found between engagement in gambling services within computer games and a history of treatment-seeking due to alcohol problems, which should be considered as an insight that expands the current scientific knowledge about how individuals involved with this activity differ from their counterparts in the general population in terms of individual characteristics and comorbidities.

## Abbreviations

DSM-5: Diagnostic and Statistical Manual of Mental Disorders, Fifth Edition
GAS: Gaming Addiction Scale
K6: Kessler Psychological Distress Scale
PGSI: Problem Gambling Severity Index

## References

[1] American Psychiatric Association (2013). Diagnostic and Statistical Manual of Mental Disorders, Fifth Edition.

[2] Calado F, Griffiths MD (2016). Problem gambling worldwide: an update and systematic review of empirical research (2000-2015). J Behav Addict.

[3] Buth S, Wurst FM, Thon N, Lahusen H, Kalke J (2017). Comparative analysis of potential risk factors for at-risk gambling, problem gambling and gambling disorder among current gamblers-results of the Austrian Representative Survey 2015. Front Psychol.

[4] Dowling N, Merkouris S, Greenwood C, Oldenhof E, Toumbourou J, Youssef G (2017). Early risk and protective factors for problem gambling: a systematic review and meta-analysis of longitudinal studies. Clin Psychol Rev.

[5] Goslar M, Leibetseder M, Muench HM, Hofmann SG, Laireiter A (2019). Pharmacological treatments for disordered gambling: a meta-analysis. J Gambl Stud.

[6] Abbott M (2017). The epidemiology and impact of gambling disorder and other gambling-related harm.

[7] Langham E, Thorne H, Browne M, Donaldson P, Rose J, Rockloff M (2016). Understanding gambling related harm: a proposed definition, conceptual framework, and taxonomy of harms. BMC Public Health.

[8] Dowling NA, Cowlishaw S, Jackson AC, Merkouris SS, Francis KL, Christensen DR (2015). The prevalence of comorbid personality disorders in treatment-seeking problem gamblers: a systematic review and meta-analysis. J Pers Disord.

[9] Lorains F, Cowlishaw S, Thomas S (2011). Prevalence of comorbid disorders in problem and pathological gambling: systematic review and meta-analysis of population surveys. Addiction.

[10] Moghaddam JF, Yoon G, Dickerson DL, Kim SW, Westermeyer J (2015). Suicidal ideation and suicide attempts in five groups with different severities of gambling: findings from the National Epidemiologic Survey on Alcohol and Related Conditions. Am J Addict.

[11] Karlsson A, Håkansson A (2018). Gambling disorder, increased mortality, suicidality, and associated comorbidity: a longitudinal nationwide register study. J Behav Addict.

[12] Wardle H, Moody J, Spence S, Orford A, Volberg R British Gambling Prevalence Survey 2010. National Centre for Social Research.

[13] Meyer G, Fiebig M, Häfeli J, Mörsen C (2011). Development of an assessment tool to evaluate the risk potential of different gambling types. Int Gambl Stud.

[14] (2019). Gambling participation in 2018: behaviour, awareness and attitudes: annual report. Gambling Commission.

[15] Gainsbury SM (2015). Online gambling addiction: the relationship between internet gambling and disordered gambling. Curr Addict Rep.

[16] Gainsbury SM, Russell A, Hing N, Wood R, Lubman D, Blaszczynski A (2015). How the Internet is changing gambling: findings from an Australian Prevalence Survey. J Gambl Stud.

[17] Håkansson A, Mårdhed E, Zaar M (2017). Who seeks treatment when medicine opens the door to pathological gambling patients-psychiatric comorbidity and heavy predominance of online gambling. Front Psychiatry.

[18] Welte JW, Barnes GM, Wieczorek WF, Tidwell MO, Hoffman JH (2007). Type of gambling and availability as risk factors for problem gambling: a tobit regression analysis by age and gender. Int Gambl Stud.

[19] Gainsbury SM, Angus DJ, Blaszczynski A (2019). Isolating the impact of specific gambling activities and modes on problem gambling and psychological distress in internet gamblers. BMC Public Health.

[20] France A (2010). Towards a sociological understanding of youth and their risk-taking. J Youth Stud.

[21] Brunborg GS, Mentzoni RA, Frøyland LR (2014). Is video gaming, or video game addiction, associated with depression, academic achievement, heavy episodic drinking, or conduct problems?. J Behav Addict.

[22] (2018). Gaming disorder.

[23] Public Health Agency of Sweden (Folkhälsomyndigheten) (2018). Samband mellan dataspel och spel om pengar.

[24] Kaess M, Parzer P, Brunner R, Koenig J, Durkee T, Carli V, Wasserman C, Hoven CW, Sarchiapone M, Bobes J, Cosman D, Värnik Airi, Resch F, Wasserman D (2016). Pathological internet use is on the rise among European adolescents. J Adolesc Health.

[25] Müller KW, Janikian M, Dreier M, Wölfling K, Beutel ME, Tzavara C, Richardson C, Tsitsika A (2015). Regular gaming behavior and internet gaming disorder in European adolescents: results from a cross-national representative survey of prevalence, predictors, and psychopathological correlates. Eur Child Adolesc Psychiatry.

[26] Mihara S, Higuchi S (2017). Cross-sectional and longitudinal epidemiological studies of Internet gaming disorder: a systematic review of the literature. Psychiatry Clin Neurosci.

[27] Paulus FW, Ohmann S, von Gontard A, Popow C (2018). Internet gaming disorder in children and adolescents: a systematic review. Dev Med Child Neurol.

[28] Lemmens JS, Valkenburg PM, Peter J (2011). Psychosocial causes and consequences of pathological gaming. Comput Human Behav.

[29] Gentile D, Choo H, Liau A, Sim T, Li D, Fung D, Khoo A (2011). Pathological video game use among youths: a two-year longitudinal study. Pediatrics.

[30] Drummond A, Sauer JD (2018). Video game loot boxes are psychologically akin to gambling. Nat Hum Behav.

[31] (2018). Skin gambling: teenage Britain's secret habit. ParentZone.

[32] Zendle D, Cairns P (2019). Loot boxes are again linked to problem gambling: results of a replication study. PLoS ONE.

[33] King D, Delfabbro P, Griffiths M (2010). The convergence of gambling and digital media: implications for gambling in young people. J Gambl Stud.

[34] Gainsbury SM (2019). Gaming-gambling convergence: research, regulation, and reactions. Gaming Law Rev.

[35] (2018). Activision Blizzard announces fourth-quarter and 2017 financial results. Activision Blizzard, Inc.

[36] King DL, Delfabbro PH (2018). Predatory monetization schemes in video games (e.g. 'loot boxes') and internet gaming disorder. Addiction.

[37] Kristiansen S, Severin MC (2020). Loot box engagement and problem gambling among adolescent gamers: findings from a national survey. Addict Behav.

[38] Wardle H (2019). The same or different? Convergence of skin gambling and other gambling among children. J Gambl Stud.

[39] Li W, Mills D, Nower L (2019). The relationship of loot box purchases to problem video gaming and problem gambling. Addict Behav.

[40] von Meduna M, Steinmetz F, Ante L, Reynolds J, Fiedler I (2019). Loot boxes: a game changer?.

[41] Schwiddessen S, Karius P (2018). Watch your loot boxes! Recent developments and legal assessment in selected key jurisdictions from a gambling law perspective. Interact Entertain Law Rev.

[42] Lee D (2018). Video game gambling banned in Belgium. BBC News.

[43] Postrado L (2018). Legal definition saves loot boxes from gambling classification in France. CalvinAyre.com.

[44] Håkansson A, Henzel V (2020). Who chooses to enroll in a new national gambling self-exclusion system? A general population survey in Sweden. Harm Reduct J.

[45] Kessler RC, Barker PR, Colpe LJ, Epstein JF, Gfroerer JC, Hiripi E, Howes MJ, Normand ST, Manderscheid RW, Walters EE, Zaslavsky AM (2003). Screening for serious mental illness in the general population. Arch Gen Psychiatry.

[46] Ferris J, Wynne H (2001). The Canadian Problem Gambling Index: final report. Canadian Consortium for Gambling Research.

[47] Currie SR, Hodgins DC, Casey DM (2013). Validity of the Problem Gambling Severity Index interpretive categories. J Gambl Stud.

[48] Lemmens JS, Valkenburg PM, Peter J (2009). Development and validation of a Game Addiction Scale for adolescents. Media Psychol.

[49] Public Health Agency of Sweden (Folkhälsomyndigheten) (2018). Results from Swelogs 2018.

[50] Public Health Agency of Sweden (Folkhälsomyndigheten) (2020). Alkohol and gambling.

[51] Dowling NA, Cowlishaw S, Jackson AC, Merkouris SS, Francis KL, Christensen DR (2015). Prevalence of psychiatric co-morbidity in treatment-seeking problem gamblers: a systematic review and meta-analysis. Aust N Z J Psychiatry.

[52] Potenza MN (2014). The neural bases of cognitive processes in gambling disorder. Trends Cogn Sci.

[53] Conversano C, Marazziti D, Carmassi C, Baldini S, Barnabei G, Dell'Osso L (2012). Pathological gambling: a systematic review of biochemical, neuroimaging, and neuropsychological findings. Harv Rev Psychiatry.

